# Relationship between Biofilm-Formation, Phenotypic Virulence Factors and Antibiotic Resistance in Environmental *Pseudomonas* *aeruginosa*

**DOI:** 10.3390/pathogens11091015

**Published:** 2022-09-05

**Authors:** Payam Behzadi, Márió Gajdács, Péter Pallós, Boglárka Ónodi, Anette Stájer, Danica Matusovits, Krisztina Kárpáti, Katalin Burián, Basem Battah, Marco Ferrari, Carlo Doria, Gianfilippo Caggiari, Ameer Khusro, Stefania Zanetti, Matthew Gavino Donadu

**Affiliations:** 1 Department of Microbiology, Shahr-e-Qods Branch, Islamic Azad University, Tehran 37541-374, Iran; 2 Department of Oral Biology and Experimental Dental Research, Faculty of Dentistry, University of Szeged, Tisza Lajos krt. 63, 6720 Szeged, Hungary; 3 Department of Periodontology, Faculty of Dentistry, University of Szeged, Tisza Lajos krt. 62–64, 6720 Szeged, Hungary; 4 Department of Prosthodontics, Faculty of Dentistry, University of Szeged, Tisza Lajos krt. 62–64, 6720 Szeged, Hungary; 5 Department of Orthodontics and Pediatric Dentistry, Faculty of Dentistry, University of Szeged, Tisza Lajos körút 62–64, 6720 Szeged, Hungary; 6 Department of Medical Microbiology, Albert Szent-Györgyi Health Center, Faculty of Medicine, University of Szeged, Semmelweis utca 6., 6725 Szeged, Hungary; 7 Department of Biochemistry and Microbiology, Faculty of Pharmacy, Syrian Private University (SPU), Daraa International Highway, 36822 Damascus, Syria; 8 Department of Biomedical Sciences, University of Sassari, 07100 Sassari, Italy; 9 Orthopaedic Department, Sassari University Hospital, 07100 Sassari, Italy; 10 Centre for Research and Development, Department of Biotechnology, Hindustan College of Arts & Science, Padur, OMR, Chennai 603103, India; 11 Hospital Pharmacy, Azienda Ospedaliero Universitaria di Sassari, 07100 Sassari, Italy

**Keywords:** *Pseudomonas aeruginosa*, antimicrobial resistance, MDR, environmental, biofilm, crystal violet, motility, siderophores, One Health, non-fermenting, phenotypic assay

## Abstract

The formation of a protective biofilm by *Pseudomonas aeruginosa* (PA) is one of the hallmarks of their survival both in vivo and in harsh environmental conditions, thus, biofilm-eradication has relevance from therapeutic perspectives and for infection control. The aim of our study was to investigate the possible relationship between antibiotic resistance, biofilm-forming capacity and virulence factors in *n* = 166 PA isolates of environmental origin. Antimicrobial susceptibility testing and the phenotypic detection of resistance determinants were carried out using standard protocols. The biofilm-forming capacity of PA was tested using a standardized crystal violet microtiter plate-based method. Motility (swimming, swarming, and twitching) and siderophore production of the isolates were also assessed. Resistance rates were highest for ciprofloxacin (46.98%), levofloxacin (45.18%), ceftazidime (31.92%) and cefepime (30.12%); 19.28% of isolates met the criteria to be classified as multidrug-resistant (MDR). Efflux pump overexpression, AmpC overexpression, and modified Hodge-test positivity were noted in 28.31%, 18.07% and 3.61%, respectively. 22.89% of isolates were weak/non-biofilm producers, while 27.71% and 49.40% were moderate and strong biofilm producers, respectively. Based on MDR status of the isolates, no significant differences in biofilm-production were shown among environmental PA (non-MDR OD_570_ [mean ± SD]: 0.416 ± 0.167 vs. MDR OD_570_: 0.399 ± 0.192; *p* > 0.05). No significant association was observed between either motility types in the context of drug resistance or biofilm-forming capacity (*p* > 0.05). 83.13% of isolates tested were positive for siderophore production. The importance of PA as a pathogen in chronic and healthcare-associated infections has been described extensively, while there is increasing awareness of PA as an environmental agent in agriculture and aquaculture. Additional studies in this field would be an important undertaking to understand the interrelated nature of biofilm production and antimicrobial resistance, as these insights may become relevant bases for developing novel therapeutics and eradication strategies against PA.

## 1. Introduction

The genus *Pseudomonas* (a member of the *Pseudomonatoda* phylum as per recent taxonomic revisions) currently consist of around 300 different species, characterized by being motile, obligate aerobic, oxidase-positive, non-fermenting Gram-negative rods [1,2]. Although *P. aeruginosa* (PA) is the most commonly isolated species from both human infections and from environmental sources, other species of the genus have also been highlighted as relevant causative agents for food and pharmaceutical spoilage (e.g., *P. putida*, *P. fluorescens*), and as plant pathogens (*P. plecoglossicida*, *P. syringae* and *P. viridiflava*) and fish (*P. anguilliseptica* and *P. baetica*) [3,4,5]. *Pseudomonas* spp. are ubiquitous in the environment—although in low abundance—but their isolation correlates with rates of anthropogenic influence; these bacteria are commonly found in aquatic sources, sediments, plants and soil, in addition to man-made sites like farming regions and hospital environments [6,7]. Their universal presence in a wide variety of habitats may be owed to their non-fastidious growth requirements and their incredible adaptability to many different environmental factors [8]. This is made possible by considerable genomic plasticity (e.g., PA has a genome size of ~5.5–7 Mb) and an interconnected quorum sensing (QS) regulatory network of four pathways (*pqs*, *rhl*, *las* and *iqs*), tightly controlling metabolic processes and the expression of many virulence factors (such as toxins, enzymes, pigment, and motility) [9,10,11]. The formation of a protective biofilm by *Pseudomonas* spp. is one of the hallmarks of their survival both in vivo (against antibiotics and immune cells) and in harsh environmental conditions, thus, biofilm-eradication has relevance from therapeutic and infection control perspectives [12,13].

The emergence of multidrug resistance (MDR) in bacteria has become one of the most daunting challenges of the 21st century, due to the increasing prevalence of difficult-to-treat infections and the lack of relevant therapeutic alternatives [14,15,16]. The magnitude of the issue has also been identified by political leaders, exemplified by the recent commitment of the G7 Nations to resistance surveillance and to invest in antimicrobial research [17]. PA is an important causative agent of serious infections in hospitalized and immunocompromised individuals, often with a high mortality rate (highest among non-fermenters) [18,19]; common clinical manifestations include ventilator-associated pneumonia, bacteremia, keratitis, swimmer’s ear, urinary tract infections, catheter-associated infections and skin and soft tissue infections (often resulting from surgery or burns) [2,8,20]. MDR in PA often results from a complex interplay of intrinsic resistance mechanisms and acquired resistance to the main antibiotic groups relevant in PA infections (i.e., anti-pseudomonal β-lactams, fluoroquinolones, aminoglycosides and polymyxins) [2,21]. In the United States (according to 2017 data), MDR PA has caused ~33,000 infections and 2700 deaths among hospitalized patients, while in the EU/EEA region (according to 2016–2020 surveillance data), resistance in PA to ≥3 antimicrobials ranged between 0–47.1% [22,23]. Carbapenem-resistant PA (CR-PA) is also included as a “Priority 1: Critical” pathogen on the World Health Organization (WHO) Priority Pathogens List [24].

In recent years, many studies have been published detailing the possible correlation or co-occurrence of the antibiotic-resistant (or the MDR) phenotype and biofilm-formation in various bacterial genera [25,26]; biofilm provides a form of “adaptive” resistance against antimicrobial drugs (resulting in lower capacity for diffusion, low oxygen tension and emergence of dormant phenotypes), in addition to some researchers suggesting common regulatory mechanisms behind biofilm-production and the expression of resistance genes [27,28]. However, the presently available evidence is contradictory and is often influenced by the heterogeneity of bacterial isolates and the methods used [29]. For example, Azizi and colleagues showed that *Acinetobacter baumannii* carrying the β-lactamase PER-1 produced more extensive biofilm—compared to non-carriers—due to an advantage in the capacity of epithelial attachment, a critical factor in early biofilm-formation [30]. On the other hand, in the case of PA, Gallant and colleagues noted an inverse relationship between the carriage of the TEM-1 β-lactamase, adhesion potential and subsequent biofilm-formation [31].

In a previous in vitro study, we assessed the correlation between biofilm-forming capacity, antimicrobial resistance and the expression of phenotypic virulence factors in randomly-selected clinical PA isolates (*n* = 302) [32]; in the study, no associations were found between MDR-status of the isolates and their propensity to form biofilm. Furthermore, no relationship was seen between biofilm formation, various motility types and pigment production (with the exception of pyocyanin) in these experiments. To complement and confirm our previous findings, our aim was to investigate the possible relationship between antibiotic resistance, biofilm-forming capacity and virulence factors in PA isolates of environmental origin. Our working hypotheses—based on our previous findings—were as follows: (i) the majority of environmental PA isolates are strong biofilm-producers; (ii) MDR is not a predictor of biofilm-forming capacity; (iii) there is no significant association between biofilm-formation and the presence of other virulence-determinants.

## 2. Materials and Methods

### 2.1. Sample Size Determination

The sample size of environmental PA isolates required for this descriptive study was determined using the formula below (1), based on the methodology described by Thrusfield et al. [33], where *n* was the calculated sample size, z was the desired level of confidence (1.96), *i* was the standard sampling error (5%), and *p* was the estimated prevalence set at 10%. The minimum required sample size (*n* = 138) was increased by 20% for added contingency [34], thus the required sample size of *n* = 166 was determined.

(1) $$n=\frac{z^{2}p\left(1-p\right)}{i^{2}}$$

### 2.2. Collection of Isolates

A total of one hundred and sixty-six (*n* = 166) PA isolates were included in the study, which was obtained from strain collections of environmental origins. The bacteria included were isolated from both outdoors (e.g., surface water, sediments, soil, agricultural sources and plants) and from surfaces with high rates of human contact (e.g., handles, steel and rubber surfaces) in Sassari (Italy) and Szeged (Hungary). Environmental sampling of these isolates was carried out via established protocols, described previously [35]. As a rule of thumb, only one PA isolate per source was included [36]. During the experiments, PA ATCC 27853 (characterized by limited biofilm-production and MDR), and PA PAE 170022 (characterized by strong biofilm-production and susceptibility to antibiotics) were used as control strains, which were obtained from the American Type Culture Collection (ATCC; Manassas, VI, USA) [32]. Stock cultures were stored at −80 °C in a cryopreservation medium (700 µL trypticase soy broth + 300 µL 50% glycerol) until further use.

### 2.3. Identification of Isolates

PA isolates were re-identified to the species level before inclusion in further experiments. Identification was carried out using matrix-assisted laser desorption/ionization–time-of-flight mass spectrometry (MALDI–TOF MS), using a MicroFlex MALDI Biotyper (Bruker Daltonics, Bremen, Germany); the technical details of the mass spectrometry measurements were described previously [32]. Spectra analysis was carried out with the MALDI Biotyper RTC 3.1 software and the MALDI Biotyper Library 3.1 (Bruker Daltonics, Bremen, Germany). During analysis, a *log*(score) value was assigned to all isolates, indicating the reliability of identification: a score ≥2.30 corresponded to reliable species-level identification [37].

### 2.4. Antimicrobial Susceptibility Testing

Susceptibility testing for relevant anti-pseudomonal antibiotics was carried out using the disk diffusion method (Oxoid, Basingstoke, UK) and E-tests (Liofilchem, Roseto degli Abruzzi, Italy) on Mueller-Hinton agar plates (bioMérieux, Marcy-l’Étoile, France) including amikacin, ceftazidime, cefepime, ciprofloxacin, gentamicin, imipenem, levofloxacin and meropenem. Colistin susceptibility was performed using the broth microdilution assay in cation-adjusted Mueller-Hinton broth (Merlin Diagnostika GmbH, Bremen, Germany). Interpretation of the results was based on the standards and breakpoints of the European Committee on Antimicrobial Susceptibility Testing (EUCAST) v. 11.0 [38]. Results indicating “susceptible, increased exposure (I)” were grouped with and reported as susceptible (S) [39]. Classification of the isolates as MDR (resistance to at least one agent in ≥3 antibiotic groups) was based on Magiorakos et al. [40]. A multiple antibiotic resistance (MAR) index—ranging between 0 and 1—was calculated by dividing the total number of detected resistance to antimicrobials for each isolate by the total number of tested antimicrobials [41].

### 2.5. Phenotypic Detection of β-Lactamases

In the case of detecting ceftazidime resistance, the overexpression of AmpC β-lactamase enzymes was tested using an agar plate method, as described previously [42]. In this assay, cloxacillin (250 µg/mL in the agar base) was used, as this antibiotic is able to inhibit the effects of AmpC β-lactamases [43]. An isolate was considered positive for AmpC-overexpression if a two-fold difference in ceftazidime MICs (measured by E-tests) were observed with or without the presence of cloxacillin [42]. Isolates were screened for carbapenemase-production—if the inhibition zone diameters around meropenem disks were 23 mm ≥ (intermediate: 23–18 mm, or resistant <19 mm)—using the modified Hodge (cloverleaf) test optimized for PA, as previously described [44,45]. During the assay, 10 µg meropenem (Oxoid, Basingstoke, UK) disks were used, while *Escherichia coli* ATCC 25922 was used as an indicator organism [46].

### 2.6. Detection of Efflux Pump Overexpression Using Phenotypic Methods

Resistance-nodulation-division (RND-type) efflux pump overexpression was tested in the case of ciprofloxacin-resistant isolates, using a phenylalanine-arginine β-naphthylamide (PAβN)-based agar dilution method [47]. During the study, the concentration of PAβN was 40 µg/mL in the agar base; a two-fold decrease in ciprofloxacin MICs (measured by E-tests) in the presence of PAβN, compared to the MIC values without the inhibitor, was considered positive for efflux pump overexpression [47]. *P. aeruginosa* ATCC 27,853 was used as a control strain.

### 2.7. Biofilm Production

Biofilm-forming capacity in environmental PA was measured using a microtiter-plate (MTP) method, as described previously [48]. Briefly, overnight *P. aeruginosa* cultures (grown on Luria–Bertani [LB] agar) were inoculated into 5 mL of LB-broth and incubated overnight at 37 °C. The following day, 180 μL of LB-broth and 20 μL of bacterial suspension set at 10^6^ CFU/mL were transferred onto 96-well flat-bottomed microtiter plates to a final volume of 200 µL and incubated for 24 h at 37 °C. Following the incubation period, the supernatants were discarded, and the wells were washed three times using 200 µL of phosphate-buffered saline (pH set at 7.2). After washing, the wells were fixed with 250 μL of methanol (Sigma-Aldrich, St. Louis, MO, USA) for 10 min and stained with a 1.0% crystal violet (CV; Sigma-Aldrich, St. Louis, MO, USA) solution for 15 min. The CV dye was discarded and the wells were washed trice with purified water to remove excess stain. The wells’ contents were solubilized in 250 μL of 33% *v*/*v*% glacial acetic acid (Sigma-Aldrich, St. Louis, MO, USA), and a microtiter plate reader was used to measure absorbance at 570 nm (OD_570_). All experiments were carried out in triplicate. Interpretation of the experimental results was carried out based on the recommendations of Ansari et al.: isolates with OD_570_ < 0.12 were classified as weak/non-biofilm producers, OD_570_ = 0.12–0.24 were classified as moderate biofilm producers, while OD_570_ > 0.24 were classified as strong biofilm producers, respectively [49,50].

### 2.8. Swimming, Swarming, and Twitching Motility

Motility assays were carried out in Petri dishes containing Tryptic Soy Agar medium with different agar concentrations (0.3% for swimming motility, 0.8% for swarming motility, and 2.0% for twitching motility, respectively) [51,52]. Overnight bacterial cultures (set at a density of 10^5^ CFU/mL) were transferred into the agar medium by puncture using a pipette tip (at 1/2 depth for swimming and swarming motility and at full depth for twitching motility) [53]. After incubation at 37 °C for 24 h (swimming and swarming motility) or 48 h (twitching motility), growth zone diameters (mm) were measured; in the case of swimming and swarming motility, the measurements were made directly, while in case of twitching motility, the agar layer was removed and the bottom of the plates was stained directly with 0.01% CV solution [51,52]. All experiments were carried out in triplicate.

### 2.9. Siderophore Production

Qualitative detection of siderophore production by environmental PA isolates was carried out using the Chrome azurol S (CAS) plate assay, as described previously [54]. Briefly, CAS plates were prepared by adding CAS solution onto melted King’s B agar medium (1:15). Actively-growing cultures of PA were spot-inoculated at the centre of CAS plates. Bacterial colonies exhibiting orange halos after 72 h of incubation (at 28 ± 2 °C) were considered positive for siderophore production [54].

### 2.10. Statistical Analysis

Descriptive statistical analysis (means with ranges and percentages) was performed using Microsoft Excel 2013 (Microsoft Corp., Redmond, WA, USA). The normality of the variables was assessed using the Kolmogorov–Smirnov test. An independent sample t-test was performed to compare measurements of biofilm-formation (OD_570_ measurements) between MDR and non-MDR PA isolates. ANOVA with Tukey’s post hoc test was used to compare growth zones (for swimming, swarming and twitching motility) between different biofilm-producers. The χ^2^ test was applied to assess the relationship between biofilm formation and siderophore production. Statistical analyses were performed with SPSS software version 22.0 (IBM Corp., Armonk, NY, USA).

## 3. Results

### 3.1. Resistance Rates and Resistotypes of Environmental PA Isolates

Resistance levels detected in environmental PA isolates were as follows (in decreasing order): ciprofloxacin 46.98% (*n* = 78), levofloxacin 45.18% (*n* = 75), ceftazidime 31.92% (*n* = 53), cefepime 30.12% (*n* = 50), gentamicin 26.51% (*n* = 44), amikacin 20.48% (*n* = 34), meropenem 10.84% (*n* = 18), imipenem 9.64% (*n* = 16), and colistin 0.60% (*n* = 1; MIC > 2 mg/L). The resistotype distribution among environmental PA isolates is presented in Table 1. Overall, seventeen (I–XVII) different resistotypes were identified, with the most numerous resistotypes being VII (resistant to ciprofloxacin, levofloxacin, ceftazidime and cefepime; *n* = 15; 9.04%) and VIII (resistant to ciprofloxacin, levofloxacin, gentamicin and amikacin; *n* = 15; 9.04%). Of these respective isolates, 19.28% (*n* = 32) met the criteria to be MDR.

### 3.2. AmpC-Overexpression, Carbapenemase-Production, and Overexpression of Efflux Pumps in Environmental PA Isolates

To assess the relevance of various resistance mechanisms contributing to the resistant phenotype in environmental PA isolates, several phenotypic tests were used. Based on the cloxacillin plate test, AmpC overexpression was detected in 56.60% (*n* = 30 out of 53 isolates; 18.07% overall) of ceftazidime-resistant isolates. Carbapenemase production was detected using the modified Hodge-test: the test was positive in 28.57% (*n* = 6, out of 21 isolates meeting inclusion criteria; 3.61% overall) of cases. Overexpression of RND-type efflux pumps—ascertained in case of detected ciprofloxacin resistance—was noted in 60.26% (*n* = 47, out of 78 isolates; 28.31% overall). Simultaneous detection of resistance mechanisms was as follows: efflux pump and AmpC overexpression in n = 14 isolates, efflux pump overexpression and cloverleaf-test positivity in *n* = 1 isolate, cloverleaf-test positivity and AmpC overexpression in *n* = 1 isolate, while detection of all three mechanisms was seen in *n* = 2 isolates, respectively.

### 3.3. Biofilm-Forming Capacity and the Relationship with Phenotypic Expression of Virulence Factors

The biofilm-forming capacity of the environmental isolates was ascertained using a microplate-based assay with CV-staining, where results were expressed after spectrophotometric measurements (OD_570_). PA PAE 170022 (positive control) presented with OD_570_ values of 0.401 ± 0.089, while PA ATCC 27853 (negative control) had OD_570_ values of 0.072 ± 0.006. Based on the CV-assay, 22.89% (*n* = 38) of isolates were weak/non-biofilm producers, 27.71% (*n* = 46) were moderate biofilm producers, while 49.40% (*n* = 82) were strong biofilm producers, respectively. Based on the MDR status of the isolates, no significant differences in biofilm-production were shown among environmental PA (OD_570_ values non-MDR [mean ± SD]: 0.416 ± 0.167 vs. MDR: 0.399 ± 0.192; *p* > 0.05); the same was true when biofilm-forming capacity was compared on an individual antibiotic-level, as no significant differences were seen in OD_570_ values (data not shown).

Results of the motility assays—in the context of the biofilm-forming capacity of the isolates—are presented in Table 2. Levels of motility (expressed in mm) were highest for swarming, then swimming and twitching motility, respectively. Neither of the motility types showed significant differences based on the biofilm-production levels of the isolates (*p* > 0.05 in all cases). No significant association was shown for motility in regards to the drug resistance phenotype (swimming: non-MDR: 24.51 ± 7.25 vs. MDR: 23.06 ± 8.03; *p* > 0.05; swarming: non-MDR: 28.11 ± 6.11 vs. MDR: 26.94 ± 5.97; *p* > 0.05; twitching: non-MDR: 10.43 ± 2.43 vs. MDR: 9.78 ± 2.82; *p* > 0.05). The majority of isolates (*n* = 138; 83.13%) tested were positive for siderophore production; no statistical association was shown between biofilm-production levels and siderophore production (Table 3.)

## 4. Discussion

PA is an important causative agent of acute and chronic infections primarily in elderly, hospitalized individuals, and in patients affected by immunosuppression (e.g., antitumor therapy), underlying conditions (e.g., cystic fibrosis [CF]) or invasive medical interventions [2,20,55]. Overall mortality associated with these infections may be as high as 20–60%, which is further compounded by the increasing levels of MDR in PA [56]. The hallmarks of PA’s pathogenicity include its cell-mediated (endotoxin, flagella, pili, and adhesins) and secreted virulence determinants (type I–III secretion systems, degrading enzymes, and exotoxins) [2,57]. Biofilm may be considered another critical secreted virulence factor, which allows for the survival of their host; biofilms have a complex composition including aggregated bacteria, carbohydrates (among them, exopolysaccharides are the most important), DNA, lipids, proteins, and ions, which all have a role in binding water to this matrix [58,59]. Many environmental bacteria owe their recalcitrance to biofilms, which may provide protection against dryness, salt stress, extreme pH, temperature shocks, a scarcity of nutrients, or toxic xenobiotics [60]. Diffusion of unwanted molecules into the biofilms may be reduced 10^1^–10^6^-fold, compared to the diffusion rate around planktonic cells [61]. Based on a meta-analysis of existing studies, 75–99% of PA tested in some phenotypic biofilm-assay were biofilm-producers, out of which, 8–50% were classified as potent biofilm-forming isolates [25].

Members of *Pseudomonas* spp. have been highlighted from a “One Health” perspective [62]: on one hand, they have been implicated as possible zoonotic (avian) pathogens, while environmental isolates have also been suggested as possible reservoirs of antibiotic resistance genes [63,64]. Since then, an increasing number of reports describe the characterization of the resistance and virulence of environmental PA isolates [65]. In our present study, we aimed to assess biofilm-forming capacity in the context of MDR and other phenotypic virulence factors, based on experiments related to environmental PA isolates. Over two-thirds (77.11%) of isolates in our sample produced biofilm, while ~50% were strong biofilm-producers; interestingly, biofilm-producers in general and strong biofilm-producers were more common among clinical isolates (80.13% and 59.27%, respectively). When compared with our previous results for clinical PA isolates [32], resistance against fluoroquinolones was the most common in isolates of both origins; on the other hand, resistance against β-lactams, aminoglycosides and the prevalence of MDR isolates was considerably higher among clinical PA. Similar to the case of clinical isolates, looking for an association between biofilm production and other study correlates largely ended up in negative results [32]. MDR status or resistance to individual antibiotics did not predict biofilm-forming capacity, in addition to finding no significant differences in motility or siderophore production in isolates with different biofilm-forming capacities.

Radó et al. characterized PA isolated from hydrocarbon or polycyclic aromatic hydrocarbon-contaminated areas using a multi-locus sequence typing (MLST) scheme [66]; they have found isolates belonging to the ST-253 (belonging to the clonal complex PA14, which are high-risk clones with a propensity to become MDR) and ST-198 (associated with CF) sequence types, and seven different serotypes were detected. All isolates carried three virulence genes (*exoY*, *exoT*, and *exoA*), while many carried the additional two genes (*exoS* and *exoU*) as well. The study did not find an association between biofilm formation and drug resistance, although most of the isolates were moderate or strong producers of biofilm [66]. In the study of Kaszab et al., *n* = 44 isolates from a similar origin were included in analyses [35]; in contrast to our findings, these isolates showed higher levels of resistance against β-lactam antibiotics, while retained susceptibility for ciprofloxacin and gentamicin; 9.1% were MDR. Nine serotypes were detected, while based on pulse-field gel electrophoresis (PFGE) analyses, several environmental strains showed considerable homology with clinical isolates. 61.3% of isolates were biofilm-producers, and 79.0% carried five out of the six virulence genes tested. The virulence potential of the isolates was also tested via a *Galleria mellonella* infection model: almost two-thirds of the isolates were virulent, demonstrating a mortality rate of 75–100% in the wax moth model. Biofilm-formation had no relevant relationship with drug resistance or virulence, while the lack of motility and the lack of *exoS*/*exoU* genes led to significantly reduced virulence [35]. Adhimi et al. characterized the diversity of pseudomonads in water dams, during which they found 21 distinct species; while resistance against amoxicillin-clavulanic acid (~67%), cefoxitin (~94%), streptomycin (~58%) and fosfomycin (~64%) were high, no resistant isolates were found towards the antibiotics included in the present study. Out of the 13 virulence genes tested, 12 were found in all isolates, while the *exoU* gene was noted in 5.8% of isolates. Although the authors did not detect any Class I or II integrons in these isolates, the authors highlighted environmental *Pseudomonas* spp. as possible reservoirs of MDR genes [67]. Thomassen et al. performed the microbiological sampling of a salmon processing facility, during which a considerable number of *Pseudomonas* spp. isolates were found. A large portion of these isolates (86%) were classified as MDR, and based on whole-genome sequencing (WGS) analyses, efflux pumps may have been important contributors to phenotypic resistance [68].

The study of Liew et al. included *n* = 215 PA isolates of both clinical and environmental origins, collected over a period of thirty years [69]; resistance rates were low, 2.6%, 8.8% and 11.4% of isolates were resistant to imipenem, meropenem, and doripenem, respectively, while some resistant isolates harboured none of the virulence genes tested. The prevalence of virulence genes in environmental isolates was similar to the ones detected from clinical isolates, suggesting that aquatic environments may be potential sources of PA infections. As previously mentioned, Gallant et al. showed that the presence of a specific resistance determinant (a β-lactamase) in PA was associated with decreased biofilm-forming capacity in these isolates [31]. Similar to our studies, Eladawy et al. also did not find an association between the drug-resistant phenotype, the presence of 11 out of 13 virulence genes (with the exception of *pelA* and *phzM*), and biofilm-formation among *n* = 103 clinical PA isolates [70]. These results are also in line with the findings of Milojkovic et al., who also did not find a significant correlation between biofilm-production and other relevant correlates (e.g., virulence and AMR-genes, production of pigment, serotypes) in PA [71]. Interestingly, both the studies of Choy et al. [72] and Bahador et al. [73] have described that the presence of various virulence genes (*exoU* alone, or *exoU* and *exoS*)—but not the MDR phenotype or resistance to individual antimicrobials—was a reliable predictor of strong biofilm-forming capacity.

In contrast to our study and the findings of Gallant et al. [31], Perez et al. reported that PA isolates originating from CF patients produced a more robust biofilm, in case of the carriage of a metallo-β-lactamase [74], while Zahedani et al. described a similar positive association between biofilm-formation and the expression of efflux pumps [75]. Additionally, several studies have described a remarkable relationship between the MDR (or XDR) phenotype and the capacity to form biofilm: the studies of Abidi et al. (involving PA from eye infections [76]), Kaiser et al. (involving isolates from both hospital and environmental samples [77]) and Karami et al. (involving isolates from both hospital and environmental samples [78]) have all demonstrated that strong biofilm-formation was strongly associated with the MDR status of the isolates. In the report by Karami et al., environmental PA isolates showed considerable resistance rates to many β-lactams and aminoglycosides, but retained susceptibility to colistin [78]. There may be some mechanistic explanations for this, as Kaiser et al. noted that isolates exhibiting extensive resistance were more vulnerable to serum killing and polymorphonuclear neutrophil (PMN) killing in vitro [77]. Interestingly, while Rodulfo et al. [79] did not find an association between the MDR/XDR phenotype, biofilm-formation, and pigment production in clinical PA, the presence of the *exoU* gene (carried by 38.1% of isolates), hemolysin-production showed significant positive, while twitching motility showed negative correlation with MDR/XDR status.

For clarity, the limitations of our study must be acknowledged: firstly, the cross-sectional nature of the study—which included isolates from a wide range of sources—but may not reflect PA from other environments. The origin, genetic composition, and sampling methods may have considerable influence in these studies, often leading to differences in results [80]. Additionally, the study employed phenotypic methods only to assess the resistance rates, virulence, and biofilm production of these isolates. For example, a quantitative, spectrophotometric, plate-based method was used to quantify biofilm-production, however, the literature reports numerous, more advanced, quantitative *in vitro*, and in vivo animal models, biosensors and flow chambers that allow for the testing of biofilm-production in conditions much closer to “real life” circumstances. The interpretation of several phenotypic assays was carried out visually, thus, the description of the results may be influenced by the expertise of the researchers. The lack of molecular biological methods (PCR, MLST, sequencing) employed is a considerable limitation of the study; therefore, we do not have information regarding the genetic origin (e.g., the clonal lineage) of the isolates, the presence and expression levels of the biofilm, virulence and/or resistance genes, or the genotype-phenotype relationship in PA. As the regulation of the cellular and metabolic processes (e.g., expression of virulence factors) may differ considerably among genera, the generalization of results among different bacteria is also limited [81]. For example, during a similar biofilm-related study on environmental *Staphylococcus* spp., no correlation was observed between MDR and biofilm formation, while a significant association was found between rifampicin resistance and strong biofilm-producers [82].; Within its limitations, our study has provided additional data on the relationship between antibiotic resistance, biofilm-forming capacity, and other relevant virulence factors. Our experiments with environmental PA have confirmed our previous findings with clinical isolates [32], i.e., the MDR phenotype and/or resistance to specific antibiotics did not have a significant relationship with biofilm-forming capacity or the phenotypic expression of virulence determinants. However, as demonstrated by earlier studies described previously, evidence in this field is still inconclusive; the use of different models to test biofilm formation may considerably inform the heterogeneity of the available results [83]. The importance of PA as a pathogen in chronic and healthcare-associated infections has been described extensively, while there is increasing awareness of PA as an environmental agent in agriculture and aquaculture. Therapy of PA infections is increasingly difficult due to the increasing number of MDR isolates, the eradication of these microorganisms is further compounded by the protective biofilm, both in vivo and in natural or industrial environments [84]. Therefore, additional studies in this field would be an important undertaking to understand the interrelated nature of biofilm production and antimicrobial resistance, as these insights may become relevant bases for developing novel therapeutics and eradication strategies against PA.

## Figures and Tables

**Table 1:** Resistotype distribution and MAR indices of environmental PA isolates.

Resistotype	Resistance Patterns	MAR Index	Ratio of Isolates (n, %)	MDR
0	None	0	84 (50.60%)	non-MDR
I	CIP	0.111	3 (1.81%)	
II	CEFT	0.111	3 (1.81%)	
III	CIP, LEV	0.222	9 (5.42%)	
IV	CEFT, CEFE	0.222	1 (0.60%)	
V	CIP, LEV, CEFT	0.333	2 (1.20%)	
VI	CIP, LEV, CEFE	0.333	2 (1.20%)	
VII	CIP, LEV, CEFT, CEFE	0.444	15 (9.04%)	
VIII	CIP, LEV, GEN, AMI	0.444	15 (9.04%)	
IX	CIP, LEV, CEFT, GEN	0.444	1 (0.60%)	MDR
X	CIP, LEV, CEFT, CEFE, GEN	0.555	5 (3.01%)	
XI	CIP, LEV, CEFT, GEN, AMI	0.555	2 (1.20%)	
XII	CIP, LEV, CEFT, CEFE, GEN, AMI	0.666	3 (1.81%)	
XIII	CIP, LEV, CEFT, CEFE, IMI, MER	0.666	6 (3.62%)	
XIV	CIP, LEV, CEFT, CEFE, GEN, AMI, MER	0.777	5 (3.01%)	
XV	CIP, LEV, CEFT, CEFE, GEN, AMI, IMI	0.777	4 (2.42%)	
XVI	CIP, LEV, CEFT, CEFE, GEN, AMI, IMI, MER	0.888	5 (3.01%)	
XVII	CIP, LEV, CEFT, CEFE, GEN, IMI, MER, COL	0.888	1 (0.60%)	

Abbreviations: CIP: ciprofloxacin, CEFT: ceftazidime, LEV: levofloxacin, CEFE: cefepime, GEN: gentamicin, AMI: amikacin, IMI: imipenem, MER: meropenem, COL: colistin, MDR: multidrug-resistant, MAR: multiple antibiotic resistance.

**Table 2:** Relationship between biofilm-formation and motility in environmental PA isolates.

Motility	Weak/Non-Biofilm Producers (*n* = 38)	Moderate Biofilm Producers (*n* = 46)	Strong Biofilm Producers (*n* = 82)	Statistics
Swimming motility (mm) (mean ± SD)	24.66 ± 8.96	23.87 ± 7.01	25.15 ± 6.94	n.s.
Swarming motility (mm) (mean ± SD)	27.98 ± 6.02	27.44 ± 6.43	28.76 ± 5.40	n.s.
Twitching motility (mm) (mean ± SD)	11.07 ± 3.65	10.88 ± 2.96	10.23 ± 2.13	n.s.

n.s.: not statistically significant.

**Table 3:** Relationship between biofilm-formation and siderophore production in environmental PA isolates.

Siderophore-Producers	Weak/Non-Biofilm Producers (*n* = 38)	Moderate Biofilm Producers (*n* = 46)	Strong Biofilm Producers (*n* = 82)	Statistics
Siderophore (+) *n* = 138	27	39	72	n.s.
Siderophore (−) *n* = 28	11	7	10	

n.s.: not statistically significant.

## Data Availability

All data generated during the study are presented in this paper.
